# A follow‐up study with a double‐blinded, randomized controlled vitamin D supplementation trial in patients with major depressive episode (DepFuD): A study protocol and baseline characteristics

**DOI:** 10.1002/fsn3.4417

**Published:** 2024-08-20

**Authors:** T. Mikola, S. M. Lehto, K. Honkalampi, M. Valkonen‐Korhonen, H. Koivumaa‐Honkanen, T. Tolmunen, V. Laukkanen, M. Pakarinen, A. Ruusunen

**Affiliations:** ^1^ Institute of Clinical Medicine University of Eastern Finland Kuopio Finland; ^2^ Institute of Clinical Medicine University of Oslo Oslo Norway; ^3^ R&D Department, Division of Mental Health Services Akershus University Hospital Lørenskog Norway; ^4^ Department of Psychiatry University of Helsinki Helsinki Finland; ^5^ School of Educational Sciences and Psychology, Philosophical Faculty University of Eastern Finland Joensuu Finland; ^6^ Mental Health and Wellbeing, Kuopio University Hospital Wellbeing Services County of North Savo Kuopio Finland; ^7^ IMPACT—The Institute for Mental and Physical Health and Clinical Translation, School of Medicine, Barwon Health Deakin University Geelong Australia; ^8^ Institute of Public Health and Clinical Nutrition University of Eastern Finland Kuopio Finland

**Keywords:** depression, depressive symptoms, intervention, mental health, vitamin D

## Abstract

Promising initial studies on vitamin D (VD) supplementation as an adjunct treatment for major depressive disorder (MDD) require rigorously designed randomized controlled trials (RCTs). We aim to investigate the effects of augmenting standard MDD treatment with VD supplementation and examine factors influencing the treatment outcome. This article describes the study design, measures, and baseline characteristics. This multicenter RCT compares the efficacy of a six‐month VD intervention at 100 micrograms/day versus 10 micrograms/day (μg/day) (4000 IU (international units)/day vs. 400 IU/day) added to a standard treatment in outpatients aged 20–61 years with MDD. The primary outcome is change in the Montgomery–Åsberg Depression Rating Scale (MADRS) score. Secondary outcomes are other indicators of mental health and functionality (BDI, SOC, 15‐D, PSS10, LS‐4, LOT‐R, YSQ‐S2‐extended, CORE‐OM, TAS‐20, BRCS, TADS, SCL‐90, DIAD, GAF), and circulating biomarkers. Intervention assessments are conducted at baseline, 3, and 6 months, and follow‐ups at 18 months and 6 years post‐baseline. The baseline sample had 319 subjects (74% women; median age 31 (inter‐quartile range (IQR) 15), mean MADRS score 21.38 (SD 6.04)), with 281 assigned to the RCT. At present, the study continues as a follow‐up study. DepFuD project will provide extensive information regarding the potential benefits of VD and enables to identify various biopsychosocial depression‐associated risk factors.

## Graphical abstract

The DepFuD project is a multicenter randomized controlled trial (RCT) investigating the effects of a six‐month vitamin D (VD) supplementation as an adjunct treatment for major depressive disorder (MDD). The study, which includes 319 subjects at baseline, assesses changes in depression rating scales and other mental health indicators, with follow‐ups at 18 months and 6 years. The project aims to provide extensive information about the potential benefits of vitamin D and identify various biopsychosocial depression‐associated risk factors.

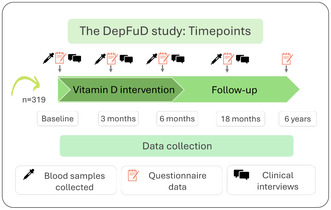

## 1 INTRODUCTION

### 1.1 Background and rationale

Major depressive disorder (MDD) is a significant mental disorder affecting an estimated 6% of adults annually with a lifetime risk of 15%–18% (Malhi & Mann, 2018). Depression is more common in women than men and more common in older than younger adults (World Health Organization, 2023). Compared to matched controls, somatic comorbidity and healthcare utilization are accentuated in people with MDD diagnosis (Steffen et al., 2020). MDD impairs psychosocial functioning, quality of life (Yang et al., 2022), and the prognosis of comorbid chronic diseases (Saint Onge et al., 2014).

Despite available treatments, many patients with MDD remain untreated due to social stigma and lack of trained healthcare providers, especially in low‐ and middle‐income countries (World Health Organization, 2023). In addition, resource scarcity may result in a greater reliance on pharmacological over psychological treatments (Cipriani et al., 2018). Antidepressants are an effective treatment for correctly diagnosed MDD (Cipriani et al., 2018), but many patients require multiple trials to achieve sufficient therapeutic response, and relapses are common (Rush et al., 2006). The heterogeneity of MDD coupled with an incomplete understanding of its pathophysiology hinders the development of more effective treatments (Malhi & Mann, 2018). Thus, research on novel treatment interventions and predictors of good treatment response is warranted.

The etiology of MDD is considered to be a combination of environmental, psychological, genetic, and biological factors (Lopresti et al., 2013). Depressive symptoms are linked to dysregulated inflammatory pathways, stress‐induced abnormal lipid and glucose metabolism (Berk et al., 2013), and a complex bidirectional relationship between endogenous inflammation and depression is suggested (Giollabhui et al., 2021). MDD is associated with compromised neurogenesis and neuronal plasticity in the brain and with hypothalamic–pituitary–adrenal (HPA) axis dysfunction (Willner et al., 2013). Dysregulated physiological pathways are influenced by stress, life events, and lifestyle factors, such as diet, physical activity, and sleep. In turn, the onset, progression, and treatment of depression can be shaped by these lifestyle factors (Lopresti et al., 2013).

Emerging evidence suggests that diet quality and nutritional components, such as vitamin D (VD), may impact the risk and progression of depressive disorders (Marx et al., 2021; Opie et al., 2017). In the central nervous system (CNS), VD is proposed to influence brain plasticity and neurotransmitter activity (Rejnmark et al., 2017), and antagonize the chronic stress effects on hippocampal neuronal differentiation and glucocorticoid receptor function (Cui & Eyles, 2022). Furthermore, human immune responses are promoted by 1,25‐dihydroxyvitamin D3 (1,25(OH)2D3) and affected by the bioavailability of circulating 25‐hydroxyvitamin D3 [25(OH)D] (Adams & Hewison, 2012).

Low 25(OH)D levels have been linked to depression in cross‐sectional studies (Anglin et al., 2013). For example, the Canadian Network for Mood and Anxiety Treatments (CANMAT) 2023 Update on Guidelines for the Management of Adults with MDD suggests patient‐specific determination of vitamin D levels in the differential diagnosis if deficiency is suspected (Lam et al., 2024). However, the current CANMAT guidelines do not recommend vitamin D supplementation as an adjunct treatment for MDD (Lam et al., 2024). Recent meta‐analyses of randomized controlled trials (RCTs) conducted in various healthy and clinical populations suggest that VD supplementation may improve depressive symptoms compared to placebo (Cheng et al., 2020; Mikola et al., 2022) and potentially reduce incidence of depression (Xie et al., 2022). These findings, due to high heterogeneity and potential publication bias, require further validation from well‐documented, long‐term, high‐quality RCTs with a follow‐up. This article presents the study design and baseline characteristics for the trial: “A follow‐up study with a double‐blinded, randomized controlled vitamin D supplementation trial in patients with major depressive episode (DepFuD)” [ClinicalTrials.gov: NCT02521012].

### 1.2 Objectives

The primary aim of the DepFuD clinical RCT is to compare the effect of a six‐month (“higher‐dose”) 100 μg (4000 IU (international units)) of VD (cholecalciferol)/day supplementation vs. (“lower‐dose”) 10 μg/day (400 IU) supplementation on depression treatment response. Secondary aims of the RCT include exploring the effect of VD supplementation on depression‐related inflammatory markers and long‐term recovery from depression. The main RCT hypothesis is that higher‐dose VD recipients will experience greater symptom reduction than lower‐dose VD supplementation recipients over 6 months. The secondary hypothesis is that depression‐related inflammatory markers differ between the RCT groups at the end of the supplementation. In addition, higher‐dose supplementation recipients are hypothesized to have better long‐term recovery from depression compared to lower‐dose supplementation recipients. The DepFuD follow‐up study also aims to identify biopsychosocial profiles predicting depression treatment response, despite VD intervention. Overall, the DepFuD project hypothesizes to be able to identify new group‐level biopsychosocial profiles predicting an individual's (a) positive treatment response in depression, (b) work ability, (c) overall mental health (including subjective wellbeing), and (d) personal and external resources.

### 1.3 Trial design

DepFuD starts with a 6‐month clinical trial using a double‐blinded, parallel‐group, randomized controlled superiority design among psychiatric adult outpatients with a 1:1 allocation ratio. The follow‐up will continue up to 6 years after the trial baseline. DepFuD employs a multidisciplinary approach, integrating psychiatry, epidemiology, public health science, physiology, and biomedicine.

### 1.4 Trial registration

The trial is registered in ClinicalTrials.gov (Identifier: NCT02521012) on August 13, 2015. All items from the World Health Organization Trial Registration Data Set can be found in the protocol and in the Table S1.

### 1.5 Protocol version

Issue date: May 2024. This is the first open access version of the study protocol, edited according to the Standard Protocol Items: Recommendations for Interventional Trials (SPIRIT) guidelines (Chan et al., 2013). See Section 3.2 for protocol amendment details.

### 1.6 Roles and responsibilities

The DepFuD project, coordinated by Kuopio University Hospital (KUH), Department of Psychiatry (Wellbeing Services County of North Savo), is led by the core group including AR, SML, KH, MVK, HKH, VL, TT, and the current corresponding investigator MP. The core group meets to make decisions regarding the research direction when needed. The project investigators are handling all aspects of the trial management without other formal groups involved in trial oversight, such as a steering committee, endpoint adjudication committee, or data management team. Formal committees were not deemed necessary due to the standard format study design and Finnish ethics regulations. See Section 2.14.1 Data monitoring for the rationalization of the lack of data monitoring committee. There are no sponsors.

## 2 METHODS

### 2.1 Study setting

#### 2.1.1 Involved centers

Wellbeing Services County of North Savo, KUH: Mental Health and Wellbeing, Psychiatry outpatient clinics.

University of Eastern Finland (UEF), Kuopio, Finland: Institute of Clinical Medicine, Department of Psychiatry; School of Educational Sciences and Psychology; Institute of Public Health and Clinical Nutrition.

Wellbeing Services County of Central Finland: Central Finland Hospital Nova, Psychiatry clinic, Jyväskylä, Finland.

Wellbeing Services County of Southern Savo, Mikkeli Central Hospital, Psychiatry clinic, Mikkeli, Finland.

### 2.2 Eligibility criteria

See Table 1 for inclusion and exclusion criteria. The majority of depressed patients in the participating units were assumed to meet the inclusion criteria for the follow‐up study and the VD intervention. Subjects who did not meet the inclusion criteria for the intervention were, upon consent, included directly in the follow‐up study only.

**TABLE 1:** Inclusion and exclusion criteria for the DepFuD study.

Follow‐up study	
Inclusion criteria	Exclusion criteria
Age: 18–65 years Sex: all Patient referred to the recruitment sites for treatment of depression Mild, moderate, or severe depression, or Mild, moderate, or severe depressive episode of recurrent depression	Psychotic depression, psychotic disorder, or bipolar disorder The person's state of health does not allow participation in the study (e.g., significant impairment of vision, hearing, or motor function) Severe substance addiction and its complications in the brain and central nervous system
*Randomized vitamin D intervention trial*	
*Vitamin D intervention trial will include all subjects eligible for the follow‐up study (same inclusion and exclusion criteria apply), with the following additional exclusion criteria:*	
Disease affecting vitamin D metabolism (sarcoidosis, hypoparathyroidism and hyperparathyroidism, hypercalcemia, kidney stones, kidney failure, or other disease affecting kidney function) Pregnancy or breastfeeding Current use of a vitamin D supplement (more than 10 μg 400 IU per day as ≤10 μg/day may be used regularly) Current use of a calcium supplement (more than 1200 mg/day)	

### 2.3 Outcomes

#### 2.3.1 Outcomes for the VD intervention

##### Primary

Between‐group differential change from baseline to 6 months in depressive symptoms as measured by the Montgomery–Åsberg Depression Rating Scale (MADRS) (range: 0–60). The changes in mean MADRS scores are compared between the subjects randomized to the higher‐dose VD supplementation group (100 μg/4000 IU per day) and the lower‐dose supplementation group (10 μg/400 IU per day).

##### Secondary

Between‐group difference in the proportion of subjects experiencing response to depression treatment (defined as MADRS score decreased by ≥50%) from baseline to 6 months.

Between‐group difference in the proportion of subjects experiencing recovery from depression (MADRS score decreases to ≤10) (Zimmerman et al., 2004).

**FIGURE 1:**
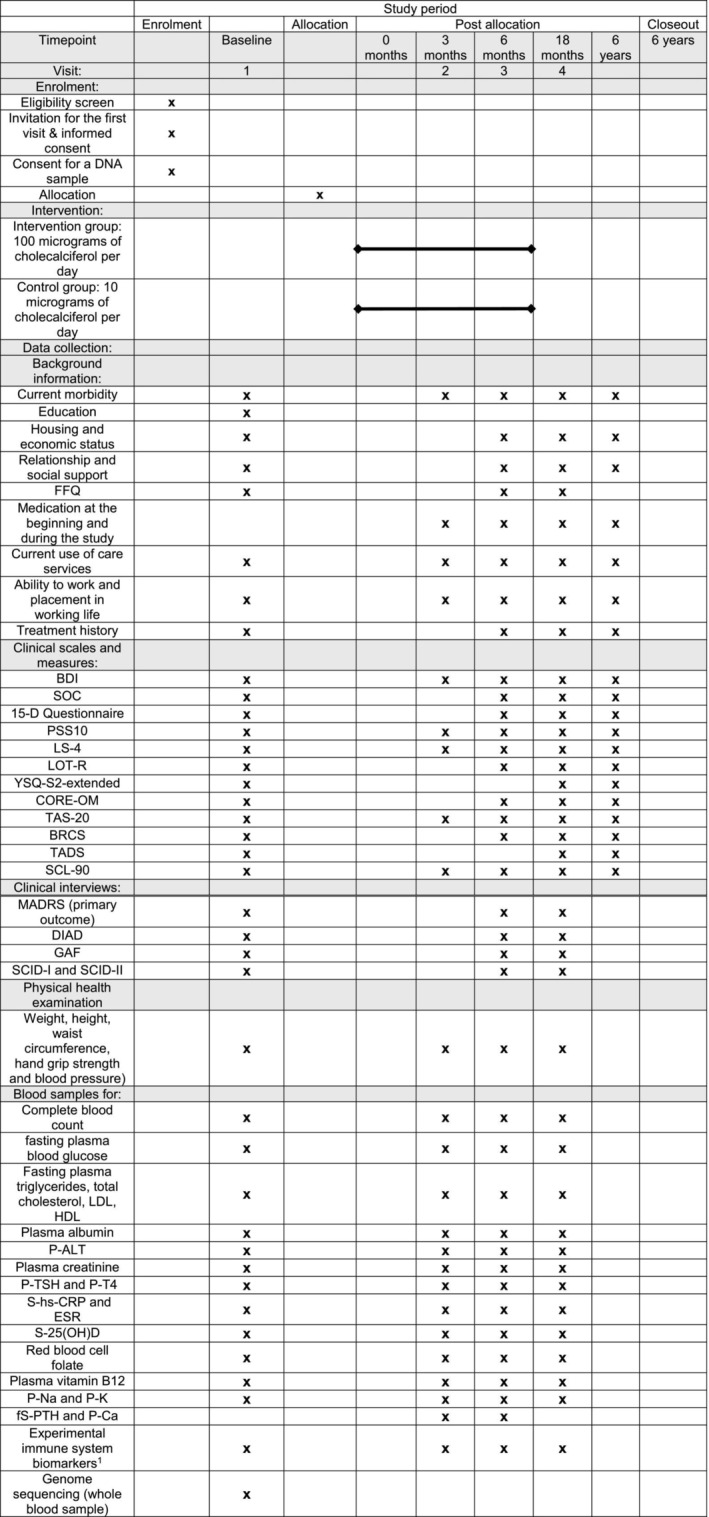
Schedule of actualized and planned patient‐related activities, collection of baseline data, and the primary and secondary outcomes during the intervention and the follow‐up in the DepFuD study. BDI, Beck Depression Inventory; BRCS, Brief Resilient Coping Scale; CORE‐OM, Clinical Outcomes in Routine Evaluation Outcome Measure; DIAD, Diagnostic Interview for Atypical Depression; ESR, erythrocyte sedimentation rate; FFQ, Food frequency questionnaire; fS‐PTH, fasting parathyroid hormone; GAF, Global Assessment of Functioning Scale; HDL, high‐density lipoprotein; LDL, low‐density lipoprotein; LOT‐R, Life Orientation Test ‐ Revised 6‐item version; LS‐4, Life Satisfaction Scale; MADRS, Montgomery–Åsberg Depression Rating Scale; P‐ALT, plasma alanine aminotransferase; P‐K, plasma potassium; P‐Na, plasma sodium; PSS10, Cohen's Perceived Stress Scale; P‐T4, plasma free thyroxine; P‐TSH, plasma thyroid‐stimulating hormone; S‐25(OH)D, serum 25‐hydroxyvitamin D; S‐Ca, plasma calcium; SCID‐I and SCID‐II, Structured Clinical Interviews for DSM‐IV; SCL‐90, Symptom Checklist‐90; S‐hs‐CRP, serum high‐sensitivity C‐reactive protein; SOC, Sense of Coherence Scale; TADS, The Trauma and Distress Scale; TAS‐20, Toronto Alexithymia Scale; YSQ‐S2‐extended, Young Schema Questionnaire S2‐extended. 1) interleukin‐1 alpha (IL‐1α), interleukin‐1 beta (IL‐1β), interleukin‐1 receptor antagonist (IL‐1ra), interleukin‐4 (IL‐4), interleukin‐6 (IL‐6), interleukin‐8 (IL‐8), interferon‐γ‐inducible protein‐10 (IP‐10), monocyte chemoattractant protein‐1 (MCP‐1), chemokine RANTES (regulated on activation, normal T‐cell expressed and secreted), transforming growth factor alpha (TGF‐α), tumor necrosis factor alpha (TNF‐α), tumor necrosis factor beta (TNF‐β), and metabolomics analyses.

Between‐group difference in the changes in other scales and measures, such as Beck Depression Inventory (BDI) and the Symptom Checklist‐90 (SCL‐90), and changes in blood markers, including inflammatory biomarkers, between baseline and 6 months. See Figure 1 for the complete list of secondary outcomes and the time points for each outcome measure. The mean/median values are compared between the higher‐dose supplementation group and the lower‐dose supplementation group at baseline and at 6 months.

#### 2.3.2 Outcomes for the follow‐up study at 18 months and at 6 years from baseline

1. Data collected with study questionnaire and blood sampling, and changes in the data during follow‐up. The complete list of outcomes and time points for each follow‐up outcome measure is presented in Figure 1. Outcomes are measured at 18 months and at 6 years from baseline. Mean/median values or proportions of subjects who experience a categorized outcome can be compared between the higher‐dose supplementation group and the lower‐dose supplementation group or based on other defined baseline or background characteristic at each time point.
2. Data from the Finnish national registers. Data obtained from national registers are linked to DepFuD with the patient's consent.
  - Diagnoses derived from the data of Finnish Institute for Health and Welfare specialized care notification register (Hilmo) and the primary care notification register (Avohilmo).
  - Diagnoses derived from the Finnish Cancer Registry.
  - Statistics Finland's—Health–Causes of death register.
  - Use of prescription medications, derived from Kela's (Finnish social insurance institution) drug registers.
  - Registers of occupational pension institutes.

### 2.4 Participant timeline

The six‐month VD RCT was implemented for each consenting subject who met the inclusion criteria for the intervention at the beginning of the study, and the study continues as a follow‐up study. The data collection for the RCT took place between November 2015 and December 2020. See Figure 1 for all actualized and planned patient‐related activities.

### 2.5 Sample size

The target sample size was derived from the VD intervention power calculation and was based on between‐group change in the MADRS score. The primary sample size calculation for DepFuD project was made in 2015 based on a small effect size of 0.15, by which the effect of the VD supplement was estimated to be at a clinically significant level, considering its benefit–risk ratio. In a two‐tailed test, with randomization ratio 1:1, alpha = 0.05, and power = 0.95, a total of 1211 individuals per intervention group should have been recruited to the study (a total of 2422 subjects). With accounting for attrition of 25%, a total of *n* = 3028 subjects would have been needed.

The target sample size was adjusted in 2019. According to previous literature, in adult patients with depression, an estimated minimal clinically important change in MADRS score during treatment was 1.6–1.9 points (Duru & Fantino, 2008). For DepFuD, a minimum of 4‐point improvement in MADRS score was considered as clinically important. With alpha = 0.05, power = 0.90, a between‐group difference of 4 units for MADRS mean, and a MADRS standard deviation (SD) = 12, a total of 382 subjects (191 per group) are required. Calculation was performed with PASS (Power Analysis and Sample Size) Software (NCSS, LLC., Kaysville, Utah) by an experienced statistician. Considering an estimated attrition of 25%, the study aimed to recruit a total of *n* = 478 subjects. The between‐group difference in MADRS mean values, which guided the determination of target sample size, is analyzed as the primary outcome, while response to treatment (defined as usual by arbitrary ≥50% MADRS score decrease from baseline) serves as a secondary outcome.

### 2.6 Recruitment

DepFuD subjects are patients from the KUH Specific Catchment Area, who were referred to the recruitment sites' (KUH Department of Psychiatry, Central Finland Hospital Nova, and Mikkeli Central Hospital) outpatient clinics for treatment of depression. The recruitment period spanned from November 2015 to June 2020. When a referral arrived, the personnel evaluated whether the patient met the inclusion criteria (Table 1). Potential study participants were evaluated by several clinicians in different centers, and the exact number of patients assessed for eligibility was unknown. If deemed suitable based on the referral, the patient received a plain language statement of the DepFuD study along with an invitation for the initial appointment. Patient could pose questions to the researchers before deciding to participate and providing an informed, written consent. The consenting subjects were assigned, either to the intervention or to the follow‐up study by the study nurse, following inclusion and exclusion criteria (Table 1).

### 2.7 Allocation

The process of allocation was independent of the enrollment personnel, and no personnel outside the research group took part in the allocation. The allocation sequence was created by AR with computer‐generated random numbers, and the coding for allocation was concealed in a sealed envelope, until intervention analyses were performed. Subjects who had given their consent and who were found suitable for the VD intervention were randomized to either the (1) higher‐dose VD supplementation group (100 μg/4000 IU of cholecalciferol per day) or the (2) lower‐dose VD supplementation group (10 μg/400 IU of cholecalciferol per day) (control group). Randomization was performed with stratified block randomization with a chosen block size of 10, which aimed to allocate the subjects to the intervention groups as evenly as possible. Randomization was conducted after the first study visit baseline examinations. After allocation to the RCT, participants who reported using a VD supplement outside the RCT were asked to discontinue the use.

### 2.8 The intervention (an RCT) and the follow‐up

The study nurse administered a six‐month daily dose of VD supplement to RCT‐assigned subjects. The VD supplement was advised to be used daily for 6 months, regardless of the season. Prohibited parallel supplements are listed in Table 1. See Section 2.14.2 Harms for criteria for discontinuing the allocated intervention. No other criteria were specified for modifying allocated interventions. During the RCT, subjects had study visits at three and 6 months from baseline. Adherence was monitored by asking participants to return used tablet jars at 6 months and by counting the number of unused tablets.

After the six‐month RCT, DepFuD continues as a clinical follow‐up study with patients with depression. The follow‐up includes filling in questionnaire (18 months and 6 years after baseline), one interview (18 months after baseline), and a blood sampling visit (18 months after baseline). Standard clinical treatment, including the use of any psychiatric medication, is provided, regardless of the RCT or the follow‐up participation.

According to the Finnish nutrition recommendations, for adults, the lower limit of adequate total intake of VD supplementation is 10 μg/400 IU per day and the upper limit of safe intake is set at 100 μg/4000 IU per day (Finnish Food Authority, 2020). The selection of the comparator dose that is nationally recommended for the most population enables a safe and ethical assessment of potential dose responses.

### 2.9 Blinding (masking)

The study participants, the study nurse, the care providers at the recruitment sites, and data analysts were blinded to allocation during the RCT. Each participant had an anonymous identification number. VD containers and all VD tablets (produced by Galena Pharma Ltd) were of identical appearance and were administered only after they had been irreversibly assigned to the participant. During the trial, the participants were not aware of their blood VD concentrations, which could indicate their assigned intervention. The researchers analyzing the primary outcome did not have access to the data until the RCT ended. The study nurse informed the attending physician in the case of abnormal plasma calcium (P‐Ca) or serum parathyroid hormone, possibly indicating pathological VD metabolism. Side effects or toxicities were unlikely, as the upper intake level of safe use for adults was set at 100 μg of vitamin cholecalciferol/ergocalciferol per day (4000 IU/day) (European Food Safety Authority, 2016). There were no specific requirements for circumstances under which unblinding subject's allocated intervention during the trial would have been permissible but if required, the corresponding investigator would have had access to group allocations.

### 2.10 Data collection, management, and analysis

Study data are collected through self‐administered paper or online questionnaires, clinical interviews, health examinations, and blood samples. Subjects were also asked for their consent to the use of their medical record data for the study. See Figure 1 for the schedule for the data collection and for the complete list of used clinical scales and measures, clinical interviews, health examinations, and analyzed biomarkers. There are no specific plans to minimize loss to follow‐up. Separate data were not collected from those who refused to participate in the study, and DepFuD data collection ends if subject discontinues participation.

The clinical scales, measures, and interviews used are internationally validated and established. A description of the study instruments along with their expected reliability and validity is presented in Table 2. Preexisting Finnish translations are used. Subjects had the opportunity to fill out the study questionnaire at their psychiatric care unit at baseline, 3, and 6 months. At 18 months, subjects received mailed questionnaires with an information letter and a return envelope. At the 6‐year follow‐up, subjects can choose whether to fill out either the mailed questionnaire or an online questionnaire. The study nurse conducted health examinations at baseline, 3 months, 6 months, and 18 months, measuring weight, height, waist circumference, hand grip strength, and blood pressure. Blood samples were taken at these same intervals.

**TABLE 2:** The description of the DepFuD study instruments along with their expected reliability and validity.

Instrument	Description
Clinical interviews	
Montgomery–Åsberg Depression Rating Scale (MADRS)	A 10‐item diagnostic questionnaire with adequate inter‐rater reliability and validity to measure the severity of depressive symptoms in patients with mood disorders. Developed to be sensitive to change in depressive symptoms and to differentiate between responders and nonresponders to antidepressant treatment. Each item is rated 0–6 and items are summed (overall score range 0–60, higher score indicating more severe depression) (Montgomery & Åsberg, 1979). A score decrease to ≤10 is defined as a remission and a score decrease by 50% from baseline is defined as a response to treatment (Zimmerman et al., 2004).
Diagnostic Interview for Atypical Depression (DIAD)	A structured interview for identifying the symptoms of atypical depression (mood reactivity, hypersomnia, leaden paralysis, hyperphagia, and rejection sensitivity) (Terman et al., 1998a). Each item is scored according to the scoring instructions of the instrument (Terman et al., 1998b). Adequate reliability and validity. Recommended instrument for confident assessment of depression with atypical features (Terman et al., 2003)
Global Assessment of Functioning Scale (GAF)	An interviewer‐used scale to rate subjectively the social, occupational, and psychological functioning of an individual on a hypothetical continuum of mental health and illness (scale range 1–100, lower score indicating more disablement and more severe symptoms within the dimension that is most adversely affected) (American Psychiatric Association, 2000). Widely used in clinical practice, despite rather poor inter‐rater reliability and poor discriminant validity with disease severity and physical limitations in a clinical setting for outpatients with depression (Grootenboer et al., 2012).
Structured Clinical Interview for DSM (SCID‐I and SCID‐II) edition DSM‐IV	A semi‐structured interview for making psychiatric diagnoses according to the diagnostic criteria from the Diagnostic and Statistical Manual of Mental Disorders (DSM‐IV). SCID‐IV for DSM‐IV has been superseded by SCID‐5 for DSM‐5, but SCID‐IV is still usable for research purposes. Following the multiaxial organization of DSM‐IV, SCID‐I is used for collecting information for Axis I disorders (major mental disorders) and SCID‐II for Axis II disorders (personality disorders) (Columbia University, Department of Psychiatry, 2018). Adequate psychometric inter‐rater and test–retest reliability for both Axis I and Axis II disorders (Zanarini et al., 2000).
Clinical scales and measures	
Beck Depression Inventory (BDI)	A 21‐item self‐reported scale for the measurement of cognitive and somatic aspects of depression and symptom intensity (scale range 0–63, higher score indicating more intense symptoms) (Beck, 1961). A good discriminant validity as a measure of depression and to reflect the severity and time frame of depressive symptoms in a Finnish population (Aalto et al., 2012).
Sense of Coherence Scale (SOC)	A 13‐item self‐reported questionnaire to assess life‐views; comprehensibility, manageability, and meaningfulness, and abilities to maintain and develop health. Items, part of which (1, 2, 3, 7, and 10) are negatively formulated, are rated with a 7‐point Likert scale with response options relevant to each item (score range 7–91, higher scores indicate greater levels of sense of coherence) (Antonovsky, 1993). High structural validity and high temporal stability in a population‐based Finnish cohort (Feldt et al., 2007).
15‐D Questionnaire	A self‐administered questionnaire to assess the different dimensions of current health‐related quality of life (mobility, vision, hearing, breathing, sleeping, eating, communication, elimination, usual activities, mental function, discomfort and symptoms, depression, distress, vitality, and sexuality). Each of the 15 items is scored on a 5‐point scale (1 indicating the best and 5 the worst possible situation). The points are used to calculate both an overall index value and a profile scoring of the dimensions on a scale of 0–1 (0 = dead, 1 = perfect health). Index value is calculated by weighting the questions with coefficients that describe the values of the population. The SPSS syntax for deriving 15‐D scores and replacing missing 15‐D data was obtained from the author. A generalizable, valid, and reliable instrument with a good discriminatory power in Western‐type societies (Sintonen, 2001).
Cohen Perceived Stress Scale (PSS10)	A 10‐item self‐reported scale to measure the degree to which situations in one's life are appraised as stressful during the last month. Score is obtained by reversing responses (0 = 4, 1 = 3, 2 = 2, 3 = 1, and 4 = 0) to the four positively stated items (4, 5, 7, and 8) and then summing across all scale items (score range 0–40, higher score indicating more stress) (Cohen & Williamson, 1988). Psychometrically validated for assessment of perceived stress in Swedish and similar populations (Nordin & Nordin, 2013).
Life Satisfaction Scale (LS‐4)	A 4‐item Allardt's self‐reported Likert scale to measure life satisfaction (range 4–20; satisfied 4–6; intermediate 7–11; and dissatisfied 12–20) with three life assessments in 5‐point scale (happiness, interestingness, and easiness of life) and one assessment in 4‐point scale (perceived loneliness). A well‐accepted and successfully used instrument for screening, monitoring, and predicting subsequent mental health among e.g. depressive patients and general populations (Koivumaa‐Honkanen et al., 2011).
Life Orientation Test‐Revised (LOT‐R)	A 6‐item self‐reported scale (without its filler items) to assess the optimistic and pessimistic dimensions of one's expectations of the future. Items are rated on a 5‐point scale. After reversing the scoring (0 = 4, 1 = 3, 2 = 2, 3 = 1, and 4 = 0) for the three negatively worded items (2, 4, and 5), item scores are totaled to yield an overall score (scale range 0–24, higher score indicating greater overall optimism) (Scheier et al., 1994). The scale has been translated and used in a Finnish population (Saari, 2019). An accurate tool for assessing individual differences on the pessimism–optimism continuum (Chiesi et al., 2013).
Young Schema Questionnaire (YSQ‐S2‐extended)	A 90‐item YSQ‐S2‐extended measures 18 early maladaptive schemas grouped under five schema domains: disconnection and rejection (includes five schemas), impaired autonomy (four schemas), impaired limits (two schemas), other‐directedness (three schemas), and overvigilance and inhibition (four schemas). The questionnaire contains 90 self‐statements (five for each schema), which respondents are asked to rate on a Likert scale ranging from 1 (completely untrue of me) to 6 (describes me perfectly). Scores for each early maladaptive schema subscale are based on the mean of the five schema statements. Total scores for schema domains are calculated as the total sum of the schema scores for each domain (Young, 1998; Young & Brown, 1994). The factor structure and psychometric properties of the Finnish version of the YSQ‐S2‐extended have been established (Saariaho et al., 2009).
Clinical Outcomes in Routine Evaluation Outcome Measure (CORE‐OM)	A 34‐item nondiagnosis‐specific self‐reported instrument with good reliability, validity, and sensitivity to change. Designed to monitor changes in psychiatric patients through the course of outpatient treatments. The maximum score is 136 and the mean score is obtained by dividing the number of total points by the number of answered questions (higher scores indicate more problems) (Evans et al., 2002). The Finnish version of CORE‐OM is psychometrically sound and is recommended for use in mental health settings (Honkalampi et al., 2017).
Toronto Alexithymia Scale (TAS‐20)	A 20‐item self‐administered questionnaire for screening the three components of alexithymia: difficulty identifying feelings, difficulty describing feelings, and externally oriented thinking. Items, five of which are negatively keyed, are rated with a 5‐point Likert scale and the score is the sum of responses to all 20 items. Cut‐off scoring: ≤51 = non‐alexithymia, ≥61 = alexithymia, and scores of 52 to 60 = possible alexithymia. In addition, the score for the three alexithymia components is calculated by using factor handling (Bagby et al., 1994). The questionnaire has been validated in several populations and languages, including Finnish (Joukamaa et al., 2001).
Brief Resilient Coping Scale (BRCS)	A 4‐item self‐reported measure designed to capture tendencies to cope with stress in a highly adaptive manner. Each item is scored from 1 to 5 (scale range 4–20, higher score indicating higher resilient coping). Developed originally in samples of adults with rheumatoid arthritis. Adequate internal consistency and test–retest reliability (Sinclair & Wallston, 2004).
The Trauma and Distress Scale (TADS)	The Finnish version of a 43‐item self‐reported questionnaire for assessment of multiple types of childhood trauma and distressing experiences. Items are rated with a 5‐point Likert scale (higher points indicate more frequent adversities) and the scores for the five TADS domains (emotional neglect, emotional abuse, physical neglect, physical abuse, and sexual abuse) are calculated by summing the respective items (final score range 0–20). A valid, reliable, and clinically useful instrument for assessing retrospectively reported childhood traumatization in a Finnish population (Salokangas et al., 2016).
Symptom Checklist‐90 (SCL‐90)	A 90‐item self‐reported questionnaire designed to measure self‐reported symptom intensity on nine different subscales (somatization, obsessive–compulsive, interpersonal sensitivity, depression, anxiety, hostility, phobic anxiety, paranoid ideation, and psychoticism). Items are rated on a 5‐point Likert scale of distress (higher points indicate higher distress) and a mean score for each subscale is calculated to be compared with population means. (Derogatis et al., 1973) A psychometrically valid and reliable instrument for psychiatric screening and outcome measuring in a Finnish population (Holi, 2003).

Clinical routine laboratory measures (fasting plasma blood glucose, fasting plasma triglycerides, total cholesterol, low‐density lipoprotein, and high‐density lipoprotein, plasma albumin, plasma alanine aminotransferase (P‐ALT), plasma creatinine, plasma thyroid‐stimulating hormone (P‐TSH) and plasma free thyroxine (P‐T4), serum high‐sensitivity C‐reactive protein (S‐hs‐CRP), erythrocyte sedimentation rate (ESR), serum 25‐hydroxyvitamin D (25(OH)D), red blood cell folate, plasma vitamin B12, plasma sodium (P‐Na) and plasma potassium (P‐K), fasting parathyroid hormone (fS‐PTH), and plasma calcium) were analyzed immediately, while experimental biomarkers and the whole blood sample for DNA analysis will be analyzed later in one batch. Data from blood sample analyses are electronic. Blood sample tests were provided by the Eastern Finland Laboratory Centre Joint Authority Enterprise (ISLAB) with the same procedures as it would occur as part of patient care without the study. Blood after centrifugation (serum and plasma) for the experimental biomarkers and the whole blood sample for DNA analysis are stored at −80°C. Experimental biomarkers in serum will be measured using multiplex biomarker panel. A DNA sample is only taken during the first blood sampling (whole blood sample).

### 2.11 Data management

DepFuD's documents are stored at KUH, Department of Psychiatry, in the password‐protected safe disk storage system provided by the KUH information administration and in the University of Eastern Finland (UEF) password‐protected personal disk storage system. Biological samples are stored in the sample freezers at KUH. Documents and biological samples are kept for 20 years, after which biological samples are physically destroyed and papers are destroyed by shredding. Data are stored and processed with Microsoft Excel (Microsoft Corporation, Redmond, WA) and IBM SPSS (IBM Corp., Armonk, NY) software. Data collected by paper forms and data obtained from blood samples are entered into the database by the study nurse and research assistants. The study questionnaires are checked for unanswered items, which, if needed, are completed with assistance from the study nurse. Range checks are performed during analyses, and identified errors are corrected, if possible. Researchers and collaborators are only provided with data that do not contain personal identification information. Data are treated as pseudo‐anonymous, with each subject assigned an identification number for the DepFuD project. The study's data are not attached to medical records, except for Structured Clinical Interviews for DSM‐IV (SCID‐I) and (SCID‐II) diagnoses. DepFuD data are not released outside the research group without a separate written data usage agreement between the research group and the external researcher. Biological samples can be analyzed anonymously also outside of Finland (countries of European Union (EU) and other countries where legislation guarantees adequate privacy protection).

### 2.12 Special considerations related to genetic research

Subjects received a separate information and consent form for taking a DNA sample and using it for research after reading the plain language statement. DNA samples are stored frozen at KUH Mental Health and Wellbeing for up to 20 years, after which further storage and analyses require a new Research Ethics Committee permit. DNA samples and other biological samples are disposed of as hazardous waste by incineration, preventing outsider access to identification information. The data obtained through the DNA sample are processed and examined only as pseudo‐anonymous coded materials. Thus, individual genetic results will not be shared with the subject or anyone else. The genetic information generated by DepFuD will be primarily used for scientific research. If the study also generates applied information suitable for therapeutic or diagnostic purposes in cooperation with industry, the operation will take place in accordance with the regulations of KUH. The DNA samples are examined for genetic characteristics that may be linked to depression or VD metabolism. The significance of some genetic characteristics is currently unknown, but future genetic research results may guide the final examinations of the stored DNA samples.

### 2.13 Statistical methods

Statistical analyses will be conducted with current versions of appropriate mainline statistical software: IBM SPSS Statistics for Windows (IBM Corp., Armonk, NY), R with suitable packages (R Foundation for Statistical Computing, Vienna, Austria), Stata Statistical Software (StataCorp, TX), or SAS software suite (SAS Institute Inc., NC). Continuous baseline and longitudinal data will be expressed as means with SDs or as medians with inter‐quartile ranges (IQRs)/ranges, whereas categorical variables will be expressed as proportions. Confidence intervals (95% CIs) will be calculated for risk ratios, for risk differences for categorical variables, and for mean differences for continuous variables. Baseline data will be analyzed cross‐sectionally for factors correlated with symptoms of depression. In cross‐sectional and longitudinal analyses e.g. analysis of variance (ANOVA), logistic or linear regression analysis, mixed models, and, if necessary, Cox regression analysis based on registry data will be utilized.

For the primary outcome, an intention‐to‐treat analysis will be performed for all randomized subjects who attended to all study visits during the intervention and completed study questionnaire without missing primary outcome data. RCT participants, who reported using over 10 μg (400 IU) of VD supplement outside the study almost daily at baseline or at 6 months despite prohibition, will be excluded from intervention analyses. Additional comparative analyses can be conducted with and without these participants. Subgroup analyses for the intervention data will be based on factors like baseline VD status, age, sex, and body mass index (BMI). If multiple effect estimates are generated for a specific outcome measurement, all estimates will be reported. Follow‐up analyses will consider background characteristics and secondary outcomes. The randomness and impact of missing data will be evaluated for primary and secondary outcome analyses, and imputation methods and sensitivity analyses may be used for specific subgroup analyses. KUH and UEF statisticians will be consulted for more advanced statistical models.

### 2.14 Monitoring

#### 2.14.1 Data monitoring

This study does not have external data monitoring committee or preplanned interim analyses for early stopping (either for futility or for positive efficacy). The absence of a data monitoring committee can be justified with a relatively short intervention duration with a well‐characterized, safe, and nonexperimental drug (VD; cholecalciferol). In Finland, the Research Ethics Committee does not require a data monitoring committee in dietary supplement interventions. The condition of the subjects in our sample of adult outpatients with nonpsychotic depression is noncritical, and the potential harm for subjects is expected to be minimal (see Section 2.14.2 Harms for stopping guidelines).

#### 2.14.2 Harms

Potential short‐term or long‐term health risks from the intervention are unlikely with the VD amounts used (10 and 100 μg/day) (European Food Safety Authority, 2016). The small tablets, sweetened with sorbitol and xylitol, were easy to consume and daily doses were not expected to have laxative effects. Research visits, giving blood samples, and filling the questionnaires cause burden to the participants. In addition, during the VD intervention, the subject had to remember to take VD supplement daily. Serum calcidiol, calcium, and parathyroid hormone concentrations were checked at baseline, 3, and 6 months to detect hypercalcemia and possible need for discontinuation. Subjects' clinical status was monitored as usual by their attending physician, and they were advised to consult their attending physician if they suspected health issues due to VD use. During the VD intervention, no unexpected harms were reported to the study group.

#### 2.14.3 Auditing

None.

## 3 ETHICS AND DISSEMINATION

### 3.1 Research ethics approval

The Medical Research Ethics Committee of Wellbeing Services County of North Savo, KUH, approved the study (ethics approval and consent number 39/2015). The study conforms to the Declaration of Helsinki (World Medical Association, 2022).

### 3.2 Protocol amendments

Protocol modifications will be submitted to the Regional Research Ethics Committee and public ClinicalTrials.gov record will be updated after protocol modifications. The updated sample size calculation and target sample size were accepted by the Research Ethics Committee in June 2020 (see Section 2.5 Sample size). In contrast to original plan, the randomization was not done separately for women and men. The time point of the final data collection is extended from original 5 to 6  years. Originally, the final follow‐up included blood samples, health examination, and clinical interviews. Due to a lack of funding, only questionnaire data will be collected at 6 years.

### 3.3 Consent or assent

The personnel at the recruitment sites obtained informed consent from the potential study participants during the first doctor's appointment. Written informed consents were then submitted to the study nurse. For more details, see Section 2.6 Recruitment and 2.11 Data management.

### 3.4 Confidentiality

See Section 2.11 Data management.

### 3.5 Declaration of interests

The authors declare that they have no competing interests. TM is funded by an UEF Doctoral Researcher position and has received grants from The Finnish Medical Foundation (grant number 4120) and The Juho Vainio Foundation (grant number 202100353). TT was supported by the Strategic Research Council within the Academy of Finland (SchoolWell, grant number 352509, work package 352511).

### 3.6 Access to data

DepFuD group researchers, as outlined in the Research Ethics Committee documentation, have access to the final dataset. Any data required to support the protocol can be supplied on request.

### 3.7 Ancillary and post‐trial care

Subjects receive standard treatment for depression during and after the intervention, with no expected harm (see Section 2.14.2 Harms) or trial participation compensation. The subjects are covered by patient injury insurance.

### 3.8 Dissemination policy

Study results will be published in international, peer‐reviewed scientific journals, reported at the group level. The individual results of the study may only be reported to the subjects if the findings are deemed to have individual‐level therapeutic significance. Current biological markers of depression have only scientific significance. Future publications will be authored by DepFuD group researchers, not professional writers. Full protocol and statistical scripts for intervention analyses will be open access. Complete participant or summary level of DepFuD dataset including sensitive information will not be shared. Metadata can be published in fairdata.fi if considered necessary.

## 4 APPENDICES

### 4.1 Informed consent materials

The plain language statement, original informed consent form, DNA sample consent form, and questionnaire in Finnish are available from the corresponding author, TM, upon reasonable request.

### 4.2 Biological specimens

See Section 2.10 Data collection, management, and analysis and 2.11 Data management.

## 5 RESULTS

### 5.1 Baseline characteristics

A total of 319 patients (302 from Kuopio, 16 from Jyväskylä, and 1 from Mikkeli) were enrolled and gave informed consent. Data from baseline assessments (self‐administered background information, self‐administered clinical scales and measures, health examinations, clinical interviews, or blood samples) are available from a total of 319 subjects. Two hundred eighty‐one subjects were assigned to the RCT and 38 chose to participate in the follow‐up study. For the RCT, subjects were randomized and double‐blinded to supplement groups 1 (142 subjects) and 2 (139 subjects).

Table 3 presents the baseline characteristics of the sample. Analyses were performed with IBM SPSS Statistics for Windows version 27. The median age was 31 years (IQR 15, range: 20–61) and 74% of the subjects were women. The most common marital status was single (50%) and 37% of the subjects were currently in a relationship. The average of full‐time schooling during life was 14.7 (SD 3.1, range: 2–26), and the highest level of completed education was most commonly middle school and vocational degree (38%) or high school and some another degree (24%). Fourteen percent of the subjects reported a completed academic degree. Forty percent of the subjects were not currently working due to various reasons, including sick leave or studying. Twenty percent of the subjects were employed full‐time, 23% were unemployed, and the rest were on different forms of pension.

**TABLE 3:** Baseline characteristics of the DepFuD participants (*n* = 319) reported as frequencies, mean (M) with standard deviation (SD), or median (Md) for non‐normally distributed data.

Background information	
Age (years), Md (25th percentile, 75th percentile)	31 (25, 40), min–max: 20–61 (missing: *n* = 3)
Men	81
Women (frequency)	236 (missing: *n* = 2)
Single	160
In relationship	116
Separated or divorced	40
Widowed (frequency)	1 (missing: *n* = 2)
Education	
Part of public or elementary school	1
Public school or part of middle school	9
Middle school or part of high school	20
Middle school and vocational education	121
High school degree	47
High school and some another degree	75
Academic degree (frequency)	44 (missing: *n* = 2)
Studied full‐time (years), M (SD)	14.7 (3.1), min–max: 2–26 (missing: *n* = 4)
Full‐time work	63
Shortened working week	10
Part‐time work	28
Unemployed	72
Unemployment pension	1
Part‐time pension	2
Disability pension	12
Employment pension	1
Out of working life for other reasons (frequency)	127 (missing: *n* = 3)
Ability to work	
Able to work	40
Reduced ability to work	164
Unable to work (frequency)	113 (missing: *n* = 2)
Personal disposable income per month in euros	
≤1000	153
1001–1500	78
1501–2500	74
2501–3500	10
3501–5000	0
>5000 (frequency)	2 (missing: *n* = 2)
Perceived economic status	
Good	16
Fairly good	125
Fairly bad	117
Poor (frequency)	58 (missing: *n* = 3)
Body mass index (BMI) (kg/m^2^): Md (25th percentile, 75th percentile)	26.33 (23.19, 31.64), min–max: 16.42–62.71 (missing: *n* = 2)
Multivitamin preparation use	
Almost daily	46
Occasionally or seasonally	72
Does not use (frequency)	199 (missing: *n* = 2)
Vitamin D supplement use	Almost daily/Occasionally or seasonally/Does not use
5–10 μg/day (200–400 IU/day)	14/27/276 (missing: *n* = 2)
11–19 μg/day (440–760 IU/day)	1/5/310 (missing: *n* = 3)
20 μg/day (800 IU/day)	14/18/285 (missing: *n* = 2)
>20 μg/day (frequencies) (>800 IU/day)	28/35/254 (missing: *n* = 2)
Fatty acid preparation (e.g., fish oil capsule) use	
Almost daily	27
Occasionally or seasonally	43
Does not use (frequency)	246 (missing: *n* = 3)
Amount of night sleep (hours): M (SD)	7.52 (1.57), min–max: 3.0–12.0 (missing: *n* = 3)
Smoked regularly for at least a year during life (frequency)	153 (missing: *n* = 3)
Current smoking (within 1 month) (frequency)	116 (missing: *n* = 3)
Alcohol use	
Does not use	32
Once a month or less	113
2–4 times a month	135
2–3 times per week	35
4 times a week or more (frequency)	2 (missing: *n* = 2)
Comorbidity	
Self‐reported diagnosed psychiatric comorbidity within 12 months:	
Personality disorder	9 (missing: *n* = 2)
Anxiety disorder	93 (missing: *n* = 4)
Eating disorder	5 (missing: *n* = 2)
Alcohol addiction	9 (missing: *n* = 2)
Drug addiction (frequency)	0 (missing: *n* = 2)
Number of self‐reported physical (and not‐elsewhere reported neuropsychiatric conditions), Md (25th percentile, 75th percentile)	1 (0, 2), min–max: 0–9
Treatment history	
Received during depression treatment history	
Drug treatment	278 (missing: *n* = 5)
Psychotherapy or talk therapy	238 (missing: *n* = 7)
Family therapy or couple therapy	57 (missing: *n* = 8)
Electroconvulsive therapy (ECT)	5 (missing: *n* = 5)
Bright light therapy	13 (missing: *n* = 4)
Music therapy	5 (missing: *n* = 5)
Art therapy	6 (missing: *n* = 4)
Exercise therapy	4 (missing: *n* = 4)
Natural medicines	16 (missing: *n* = 4)
Hypnosis therapy	1 (missing: *n* = 4)
Lifestyle guidance (frequency)	77 (missing: *n* = 6)
Duration of depressive episode (months): Md (25th percentile, 75th percentile)	12 (6, 26), min–max: 1–216 (missing: *n* = 9)
Number of depressive episodes during lifetime: Md (25th percentile, 75th percentile)	2 (1, 3), min–max: 1–12 (missing: *n* = 7)
Clinical interviews	
Montgomery–Åsberg Depression Rating Scale (MADRS): M (SD)	21.38 (6.04), min–max: 8–37 (missing: *n* = 0)
Diagnostic Interview for Atypical Depression (DIAD)	
Meets atypical depression features criteria	105 (missing: *n* = 1)
Meets DSM‐IV criteria for melancholic features	60 (missing: *n* = 1)
Meets atypical features criteria and does not meet criteria for melancholic features (frequency)	85 (missing: *n* = 2)
Global Assessment of Functioning (GAF): Md (25th percentile, 75th percentile)	52 (50, 58), min–max: 44–80 (missing: *n* = 0)
Clinical scales and measures	
Beck Depression Inventory (BDI): M (SD)	22.26 (9.66), min–max: 0–46 (missing: *n* = 8)
Sense of Coherence Scale (SOC): M (SD)	
Total score	46.89 (12.37), min–max: 21–84 (missing: *n* = 5)
Comprehensibility	17.23 (5.67), min–max: 6–33 (missing: *n* = 5)
Manageability	14.86 (4.54), min–max: 4–26 (missing: *n* = 4)
Meaningfulness	14.81 (4.71), min–max: 4–27 (missing: *n* = 4)
15‐D Questionnaire: M (SD)	0.7645 (0.0919), min–max: 0.4054–0.9815 (missing: *n* = 2)
Cohen Perceived Stress Scale (PSS10): M (SD)	22.77 (6.17), min–max: 2–36 (missing: *n* = 7)
Life Satisfaction Scale (LS‐4): M (SD)	14.09 (3.47), min–max: 5–20 (missing: *n* = 4)
Life Orientation Test‐Revised (LOT‐R) 6‐item version: M (SD)	10.05 (4.73), min–max: 0–21 (missing: *n* = 6)
Young Schema Questionnaire (YSQ‐S2‐extended): M (SD)	
Disconnection & rejection domain score	14.55 (5.20), min–max: 5.4–28.0 (missing: *n* = 6)
Impaired autonomy & performance domain score	9.46 (3.16), min–max: 4.0–19.0 (missing: *n* = 10)
Impaired limits domain score	4.84 (1.56), min–max: 2.0–11.0 (missing: *n* = 5)
Other‐directedness domain score	9.79 (2.30), min–max: 3.6–17.2 (missing: *n* = 7)
Overvigilance & inhibition domain score	13.18 (3.50), min–max: 4.6–21.6 (missing: *n* = 6)
Clinical Outcomes in Routine Evaluation Outcome Measure (CORE‐OM) Total raw score: M (SD)	54.46 (16.21), min–max: 7–105 (missing: *n* = 4)
Toronto Alexithymia Scale (TAS‐20): M (SD)	
Total score	53.67 (11.0), min–max: 22–81 (missing: *n* = 4)
Difficulty in identifying feelings factor score	19.91 (5.65), min–max: 7–33 (missing: *n* = 4)
Difficulty in describing feelings to others factor score	14.37 (4.56), min–max: 5–24 (missing: *n* = 4)
Externally oriented thinking factor score	19.38 (4.40), min–max: 8–35 (missing: *n* = 4)
Brief Resilient Coping Scale (BRCS): M (SD)	11.69 (2.91), min–max: 4–20 (missing: *n* = 4)
The Trauma and Distress Scale (TADS): Md (25th percentile, 75th percentile)	
Total score	3.6 (2.0, 6.2), min–max: 0–15.4 (missing: *n* = 6)
Emotional abuse factor score	5.0 (2.0, 9.0), min–max: 0–19.0 (missing: *n* = 6)
Physical abuse factor score	1.0 (0, 3.0), min–max: 0–20 (missing: *n* = 4)
Sexual abuse factor score	0 (0, 1.0), min–max: 0–17 (missing: *n* = 4)
Emotional neglect factor score	8.0 (5.0, 12.0), min–max: 0–20 (missing: *n* = 4)
Physical neglect factor score	4.0 (2.0, 7.0), min–max: 0–16 (missing: *n* = 4)
Symptom Checklist‐90 (SCL‐90) Total score: M (SD)	1.26 (0.56), min–max: 0.04–3.32 (missing: *n* = 3)
Blood samples	
Fasting plasma blood glucose (mmol/L), Md (25th percentile, 75th percentile)	5.4 (5.1, 5.8), min–max: 4.0–27.1 (missing: *n* = 2)
Fasting plasma triglycerides (mmol/L), Md (25th percentile, 75th percentile)	1.04 (0.72, 1.50), min–max: 0.33–5.91 (missing: *n* = 1)
Total cholesterol (mmol/L), M (SD)	4.76 (0.95), min–max: 2.6–8.6 (missing: *n* = 2)
Low‐density lipoprotein (LDL) (mmol/L), M (SD)	3.0 (0.95), min–max: 0.9–6.6 (missing: *n* = 1)
High‐density lipoprotein (HDL) (mmol/L), M (SD)	1.59 (0.44), min–max: 0.71–3.02 (missing: *n* = 1)
Plasma albumin (g/L), M (SD)	40.94 (4.07), min–max: 29.0–64.0 (missing: *n* = 2)
Serum high‐sensitivity C‐reactive protein (mg/L), Md (25th percentile, 75th percentile)	0.9 (0.4, 2.83), min–max: 0.0–30.3 (missing: *n* = 17)
Erythrocyte sedimentation rate (mm/h), Md (25th percentile, 75th percentile)	5.0 (2.0, 8.0), min–max: 2.0–37.0 (missing: *n* = 2)
Serum 25‐hydroxyvitamin D (nmol/L), M (SD)	60.96 (25.8), min–max: 14.0–183.0 (missing: *n* = 2)
Red blood cell folate (nmol/L), M (SD)	1825.91 (422.06), min–max: 1148.0–4696.0 (missing: *n* = 5)
Plasma vitamin B12 (pg/mL), M (SD)	414.78 (191.91), min–max: 88.0–1414.0 (missing: *n* = 1)
Fasting parathyroid hormone (pg/mL), M (SD)	49.92 (21.13), min–max: 5.0–183.0 (missing: *n* = 5)
Plasma calcium (mmol/L), M (SD)	2.33 (0.09), min–max: 2.09–2.89 (missing: *n* = 2)

The mean MADRS score was 21.38 (SD 6.04, range: 8–37). The median number of depressive episodes was two (IQR 2, range: 1–12), lasting a median of 12 months (IQR 20, range: 1–216). On average, subjects had 7.52 hours (SD 1.57, range: 3.0–12) of night sleep, had a median BMI of 26.33 kg/m^2^ (IQR 8.45, range: 16.42–62.71), and used alcohol mostly two to four times a month (43%) or less (36%). Forty‐eight of the subjects had smoked regularly during life and 37% reported current smoking. The mean serum 25‐hydroxyvitamin D concentration was 60.96 nmol/L (SD 25.8, range: 14.0–183.0). Table 3 shows the numbers of those who used a daily value (DV) supplement outside the study protocol at the baseline for the entire data. There were 19 participants allocated to the RCT who reported off‐protocol use of VD supplement “almost daily” at baseline (one in the category 11–19 g/day, six in the category 20 μg/day, and 12 in the category 20 μg/day or higher).

## 6 CONCLUSIONS

The DepFuD project will provide extensive information regarding the potential benefits of VD intervention in clinically depressed subjects. Multiple serum calcidiol measurements, accounting for seasonal variations, enable to assess the impact of baseline calcidiol levels on the potential benefits of VD supplementation in recovery from depression. The longitudinal study design with extensive data enables the identification of various biopsychosocial risk factors associated with different dimensions of depression and indicators of mental health.

## AUTHOR CONTRIBUTIONS

**T. Mikola:** Writing – original draft (lead); writing – review and editing (equal). **S. M. Lehto:** Methodology (equal); supervision (equal); writing – review and editing (equal). **K. Honkalampi:** Conceptualization (equal); methodology (equal). **M. Valkonen‐Korhonen:** Conceptualization (equal). **H. Koivumaa‐Honkanen:** Writing – review and editing (equal). **T. Tolmunen:** Writing – review and editing (equal). **V. Laukkanen:** Writing – review and editing (equal). **M. Pakarinen:** Writing – review and editing (equal). **A. Ruusunen:** Conceptualization (equal); methodology (equal); supervision (lead); writing – review and editing (equal).

## FUNDING INFORMATION

The DepFuD project has received funding from the Finnish state research funding (VTR). The project is to be financed entirely with research funding. The funder will not influence the study design, data management, analysis, report writing, or publication decision. DepFuD does not involve commercial partners or provide commercial benefits for the researchers. Researchers will not receive bonus funding regardless of the study results.

## CONFLICT OF INTEREST STATEMENT

Authors have no competing interests.

## ETHICS STATEMENT

The Medical Research Ethics Committee of Wellbeing Services County of North Savo, Kuopio University Hospital, approved the study (ethics approval and consent number 39/2015).

## PATIENT CONSENT STATEMENT

All participants provided informed consent for participation in the study, including a separate consent for the collection of DNA samples for research purposes.

## PERMISSION TO REPRODUCE MATERIAL FROM OTHER SOURCES

Participants decided on the use of their medical records in the study. Data from national registers are linked to the project with patient consent.

## Supporting information


Table S1

## Data Availability

The statistical scripts for intervention analyses will be published as open access or as supplementary material. Complete participant or summary level of DepFuD dataset including sensitive information will not be shared. Metadata can be published in fairdata.fi if considered necessary in the future.

## References

[fsn34417-bib-0001] Aalto, A.‐M. , Elovainio, M. , Kivimäki, M. , Uutela, A. , & Pirkola, S. (2012). The Beck depression inventory and general health questionnaire as measures of depression in the general population: A validation study using the composite international diagnostic interview as the gold standard. Psychiatry Research, 197(1), 163–171. 10.1016/j.psychres.2011.09.008 22365275

[fsn34417-bib-0002] Adams, J. S. , & Hewison, M. (2012). Extrarenal expression of the 25‐hydroxyvitamin D‐1‐hydroxylase. Archives of Biochemistry and Biophysics, 523(1), 95–102. 10.1016/j.abb.2012.02.016 22446158 PMC3361592

[fsn34417-bib-0003] American Psychiatric Association . (2000). Diagnostic and statistical manual of mental disorders, Fourth Edition, Text Revision . 10.1111/j.1743-6109.2007.00557.x 17727354

[fsn34417-bib-0004] Anglin, R. E. S. , Samaan, Z. , Walter, S. D. , & McDonald, S. D. (2013). Vitamin D deficiency and depression in adults: Systematic review and meta‐analysis. British Journal of Psychiatry, 202(2), 100–107. 10.1192/bjp.bp.111.106666 23377209

[fsn34417-bib-0005] Antonovsky, A. (1993). The structure and properties of the sense of coherence scale. Social Science & Medicine, 36(6), 725–733. 10.1016/0277-9536(93)90033-Z 8480217

[fsn34417-bib-0006] Bagby, R. M. , Parker, J. D. A. , & Taylor, G. J. (1994). The twenty‐item Toronto alexithymia scale—I. Item selection and cross‐validation of the factor structure. Journal of Psychosomatic Research, 38(1), 23–32. 10.1016/0022-3999(94)90005-1 8126686

[fsn34417-bib-0007] Beck, A. T. (1961). An inventory for measuring depression. Archives of General Psychiatry, 4(6), 561–571. 10.1001/archpsyc.1961.01710120031004 13688369

[fsn34417-bib-0008] Berk, M. , Williams, L. J. , Jacka, F. N. , O'Neil, A. , Pasco, J. A. , Moylan, S. , Allen, N. B. , Stuart, A. L. , Hayley, A. C. , Byrne, M. L. , & Maes, M. (2013). So depression is an inflammatory disease, but where does the inflammation come from? BMC Medicine, 11, 200. 10.1186/1741-7015-11-200 24228900 PMC3846682

[fsn34417-bib-0009] Chan, A.‐W. , Tetzlaff, J. M. , Altman, D. G. , Laupacis, A. , Gøtzsche, P. C. , Krleža‐Jerić, K. , Hróbjartsson, A. , Mann, H. , Dickersin, K. , Berlin, J. A. , Doré, C. J. , Parulekar, W. R. , Summerskill, W. S. M. , Groves, T. , Schulz, K. F. , Sox, H. C. , Rockhold, F. W. , Rennie, D. , & Moher, D. (2013). SPIRIT 2013 statement: Defining standard protocol items for clinical trials. Annals of Internal Medicine, 158(3), 200–207. 10.7326/0003-4819-158-3-201302050-00583 23295957 PMC5114123

[fsn34417-bib-0010] Cheng, Y.‐C. , Huang, Y.‐C. , & Huang, W.‐L. (2020). The effect of vitamin D supplement on negative emotions: A systematic review and meta‐analysis. Depression and Anxiety, 37(6), 549–564. 10.1002/da.23025 32365423

[fsn34417-bib-0011] Chiesi, F. , Galli, S. , Primi, C. , Innocenti Borgi, P. , & Bonacchi, A. (2013). The accuracy of the life orientation test–revised (LOT–R) in measuring dispositional optimism: Evidence From item response theory analyses. Journal of Personality Assessment, 95(5), 523–529. 10.1080/00223891.2013.781029 23570253

[fsn34417-bib-0012] Cipriani, A. , Furukawa, T. A. , Salanti, G. , Chaimani, A. , Atkinson, L. Z. , Ogawa, Y. , Leucht, S. , Ruhe, H. G. , Turner, E. H. , Higgins, J. P. T. , Egger, M. , Takeshima, N. , Hayasaka, Y. , Imai, H. , Shinohara, K. , Tajika, A. , Ioannidis, J. P. A. , & Geddes, J. R. (2018). Comparative efficacy and acceptability of 21 antidepressant drugs for the acute treatment of adults with major depressive disorder: A systematic review and network meta‐analysis. Lancet, 391(10128), 1357–1366. 10.1016/S0140-6736(17)32802-7 29477251 PMC5889788

[fsn34417-bib-0013] Cohen, S. , & Williamson, G. (1988). Perceived stress in a probability sample of the United States. In S. Spacapan & S. Oskamp (Eds.), The social psychology of health: Claremont symposium on applied social psychology (pp. 31–67). SAGE.

[fsn34417-bib-0014] Columbia University Department of Psychiatry . (2018). Structured clinical interview for DSM disorders (SCID). Columbia University Department of Psychiatry. https://www.columbiapsychiatry.org/research/research‐areas/services‐policy‐and‐law/structured‐clinical‐interview‐dsm‐disorders‐scid

[fsn34417-bib-0015] Cui, X. , & Eyles, D. W. (2022). Vitamin D and the central nervous system: Causative and preventative mechanisms in brain disorders. Nutrients, 14(20), 4353. 10.3390/nu14204353 36297037 PMC9610817

[fsn34417-bib-0016] Derogatis, L. R. , Lipman, R. S. , & Covi, L. (1973). SCL‐90: An outpatient psychiatric rating scale—preliminary report. Psychopharmacology Bulletin, 9(1), 13–28. 4682398

[fsn34417-bib-0017] Duru, G. , & Fantino, B. (2008). The clinical relevance of changes in the Montgomery‐Asberg depression rating scale using the minimum clinically important difference approach. Current Medical Research and Opinion, 24(5), 1329–1335. 18377706 10.1185/030079908x291958

[fsn34417-bib-0018] European Food Safety Authority . (2016). Dietary reference values for vitamin D. EFSA Journal, 14(10), 11. 10.2903/j.efsa.2016.4547 PMC7010012 32625486

[fsn34417-bib-0019] Evans, C. , Connell, J. , Barkham, M. , Margison, F. , McGrath, G. , Mellor‐Clark, J. , & Audin, K. (2002). Towards a standardised brief outcome measure: Psychometric properties and utility of the CORE–OM. The British Journal of Psychiatry, 180(1), 51–60. 10.1192/bjp.180.1.51 11772852

[fsn34417-bib-0020] Feldt, T. , Lintula, H. , Suominen, S. , Koskenvuo, M. , Vahtera, J. , & Kivimäki, M. (2007). Structural validity and temporal stability of the 13‐item sense of coherence scale: Prospective evidence from the population‐based HeSSup study. Quality of Life Research, 16(3), 483–493. 10.1007/s11136-006-9130-z 17091360

[fsn34417-bib-0021] Finnish Food Authority . (2020). Special instructions and restrictions: Vitamin D supplementation . Healthy Diet: Nutrition and Food Recommendations. Retrieved 12 July 2023. https://www.ruokavirasto.fi/en/foodstuffs/healthy‐diet/nutrition‐and‐food‐recommendations/special‐instructions‐and‐restrictions/

[fsn34417-bib-0022] Giollabhui, N. M. , Ng, T. H. , Ellman, L. M. , & Alloy, L. B. (2021). The longitudinal associations of inflammatory biomarkers and depression revisited: Systematic review, meta‐analysis, and meta‐regression. Molecular Psychiatry, 26(7), 3302–3314. 10.1038/s41380-020-00867-4 32807846 PMC7887136

[fsn34417-bib-0023] Grootenboer, E. M. V. , Giltay, E. J. , Van Der Lem, R. , Van Veen, T. , Van Der Wee, N. J. A. , & Zitman, F. G. (2012). Reliability and validity of the global assessment of functioning scale in clinical outpatients with depressive disorders: GAF in outpatients with depression. Journal of Evaluation in Clinical Practice, 18(2), 502–507. 10.1111/j.1365-2753.2010.01614.x 21223457

[fsn34417-bib-0024] Holi, M. (2003). Assessment of psychiatric symptoms using the SCL‐90 . [(Pp. 44–58) Academic dissertation, University of Helsinki, Department of Psychiatry]. https://helda.helsinki.fi/handle/10138/22453

[fsn34417-bib-0025] Honkalampi, K. , Laitila, A. , Juntunen, H. , Lehmus, K. , Piiparinen, A. , Törmänen, I. , Inkinen, M. , & Evans, C. (2017). The Finnish clinical outcome in routine evaluation outcome measure: Psychometric exploration in clinical and non‐clinical samples. Nordic Journal of Psychiatry, 71(8), 589–597. 10.1080/08039488.2017.1365378 28836472

[fsn34417-bib-0026] Joukamaa, M. , Miettunen, J. , Kokkonen, P. , Koskinen, M. , Julkunen, J. , Kauhanen, J. , Jokelainen, J. , Veijola, J. , Läksy, K. , & Järvelin, M.‐R. (2001). Psychometric properties of the Finnish 20‐item Toronto alexithymia scale. Nordic Journal of Psychiatry, 55(2), 123–127. 10.1080/08039480151108561 11802910

[fsn34417-bib-0027] Koivumaa‐Honkanen, H. , Rissanen, T. , Hintikka, J. , Honkalampi, K. , Haatainen, K. , Tarja, S. , & Viinamäki, H. (2011). Factors associated with life satisfaction in a 6‐year follow‐up of depressive out‐patients. Social Psychiatry and Psychiatric Epidemiology, 46(7), 595–605. 10.1007/s00127-010-0225-z 20428841

[fsn34417-bib-0028] Lam, R. W. , Kennedy, S. H. , Adams, C. , Bahji, A. , Beaulieu, S. , Bhat, V. , Blier, P. , Blumberger, D. M. , Brietzke, E. , Chakrabarty, T. , Do, A. , Frey, B. N. , Giacobbe, P. , Gratzer, D. , Grigoriadis, S. , Habert, J. , Ishrat Husain, M. , Ismail, Z. , McGirr, A. , … Milev, R. V. (2024). Canadian network for mood and anxiety treatments (CANMAT) 2023 update on clinical guidelines for Management of Major Depressive Disorder in adults: Réseau canadien pour les traitements de l'humeur et de l'anxiété (CANMAT) 2023: Mise à jour des lignes directrices cliniques pour la prise en charge du trouble dépressif majeur chez les adultes. The Canadian Journal of Psychiatry, 7067437241245384. Advance online publication. 10.1177/07067437241245384 PMC11351064 38711351

[fsn34417-bib-0029] Lopresti, A. L. , Hood, S. D. , & Drummond, P. D. (2013). A review of lifestyle factors that contribute to important pathways associated with major depression: Diet, sleep and exercise. Journal of Affective Disorders, 148(1), 12–27. 10.1016/j.jad.2013.01.014 23415826

[fsn34417-bib-0030] Malhi, G. S. , & Mann, J. J. (2018). Depression. The Lancet, 392(10161), 2299–2312. 10.1016/S0140-6736(18)31948-2 30396512

[fsn34417-bib-0031] Marx, W. , Lane, M. , Hockey, M. , Aslam, H. , Berk, M. , Walder, K. , Borsini, A. , Firth, J. , Pariante, C. M. , Berding, K. , Cryan, J. F. , Clarke, G. , Craig, J. M. , Su, K.‐P. , Mischoulon, D. , Gomez‐Pinilla, F. , Foster, J. A. , Cani, P. D. , Thuret, S. , … Jacka, F. N. (2021). Diet and depression: Exploring the biological mechanisms of action. Molecular Psychiatry, 26(1), 134–150. 10.1038/s41380-020-00925-x 33144709

[fsn34417-bib-0032] Mikola, T. , Marx, W. , Lane, M. M. , Hockey, M. , Loughman, A. , Rajapolvi, S. , Rocks, T. , O'Neil, A. , Mischoulon, D. , Valkonen‐Korhonen, M. , Lehto, S. M. , & Ruusunen, A. (2022). The effect of vitamin D supplementation on depressive symptoms in adults: A systematic review and meta‐analysis of randomized controlled trials. Critical Reviews in Food Science and Nutrition, 63(33), 11784–11801. 10.1080/10408398.2022.2096560 35816192

[fsn34417-bib-0033] Montgomery, S. A. , & Åsberg, M. (1979). A new depression scale designed to be sensitive to change. The British Journal of Psychiatry, 134(4), 382–389. 10.1192/bjp.134.4.382 444788

[fsn34417-bib-0034] Nordin, M. , & Nordin, S. (2013). Psychometric evaluation and normative data of the Swedish version of the 10‐item perceived stress scale. Scandinavian Journal of Psychology, 54(6), 502–507. 10.1111/sjop.12071 24118069

[fsn34417-bib-0035] Opie, R. s. , Itsiopoulos, C. , Parletta, N. , Sanchez‐Villegas, A. , Akbaraly, T. N. , Ruusunen, A. , & Jacka, F. N. (2017). Dietary recommendations for the prevention of depression. Nutritional Neuroscience, 20(3), 161–171. 10.1179/1476830515Y.0000000043 26317148

[fsn34417-bib-0036] Rejnmark, L. , Bislev, L. S. , Cashman, K. D. , Eiríksdottir, G. , Gaksch, M. , Grübler, M. , Grimnes, G. , Gudnason, V. , Lips, P. , Pilz, S. , van Schoor, N. M. , Kiely, M. , & Jorde, R. (2017). Non‐skeletal health effects of vitamin D supplementation: A systematic review on findings from meta‐analyses summarizing trial data. PLoS One, 12(7), e0180512. 10.1371/journal.pone.0180512 28686645 PMC5501555

[fsn34417-bib-0037] Rush, A. J. , Trivedi, M. H. , Wisniewski, S. R. , Nierenberg, A. A. , Stewart, J. W. , Warden, D. , Niederehe, G. , Thase, M. E. , Lavori, P. W. , Lebowitz, B. D. , McGrath, P. J. , Rosenbaum, J. F. , Sackeim, H. A. , Kupfer, D. J. , Luther, J. , & Fava, M. (2006). Acute and longer‐term outcomes in depressed outpatients requiring one or several treatment steps: A STAR*D report. American Journal of Psychiatry, 163(11), 1905–1917. 10.1176/ajp.2006.163.11.1905 17074942

[fsn34417-bib-0038] Saari, M. (2019). Mother's and their child's optimism as predictors for family functioning [(14) Master's thesis, University of Helsinki]. https://helda.helsinki.fi/handle/10138/304795?show=full

[fsn34417-bib-0039] Saariaho, T. , Saariaho, A. , Karila, I. , & Joukamaa, M. (2009). The psychometric properties of the Finnish Young schema questionnaire in chronic pain patients and a non‐clinical sample. Journal of Behavior Therapy and Experimental Psychiatry, 40(1), 158–168. 10.1016/j.jbtep.2008.07.005 18804198

[fsn34417-bib-0040] Saint Onge, J. M. , Krueger, P. M. , & Rogers, R. G. (2014). The relationship between major depression and nonsuicide mortality for U.S. adults: The importance of health behaviors. The Journals of Gerontology Series B: Psychological Sciences and Social Sciences, 69(4), 622–632. 10.1093/geronb/gbu009 24569003 PMC4049146

[fsn34417-bib-0041] Salokangas, R. K. R. , Schultze‐Lutter, F. , Patterson, P. , von Reventlow, H. G. , Heinimaa, M. , From, T. , Luutonen, S. , Hankala, J. , Kotimäki, M. , & Tuominen, L. (2016). Psychometric properties of the trauma and distress scale, TADS, in an adult community sample in Finland. European Journal of Psychotraumatology, 7(1), 30062. 10.3402/ejpt.v7.30062 27032511 PMC4816812

[fsn34417-bib-0042] Scheier, M. F. , Carver, C. S. , & Bridges, M. W. (1994). Distinguishing optimism from neuroticism (and trait anxiety, self‐mastery, and self‐esteem): A reevaluation of the life orientation test. Journal of Personality and Social Psychology, 67(6), 1063–1078. 10.1037/0022-3514.67.6.1063 7815302

[fsn34417-bib-0043] Sinclair, V. G. , & Wallston, K. A. (2004). The development and psychometric evaluation of the brief resilient coping scale. Assessment, 11(1), 94–101. 10.1177/1073191103258144 14994958

[fsn34417-bib-0044] Sintonen, H. (2001). The 15D instrument of health‐related quality of life: Properties and applications. Annals of Medicine, 33(5), 328–336. 10.3109/07853890109002086 11491191

[fsn34417-bib-0045] Steffen, A. , Nübel, J. , Jacobi, F. , Bätzing, J. , & Holstiege, J. (2020). Mental and somatic comorbidity of depression: A comprehensive cross‐sectional analysis of 202 diagnosis groups using German nationwide ambulatory claims data. BMC Psychiatry, 20(1), 142. 10.1186/s12888-020-02546-8 32228541 PMC7106695

[fsn34417-bib-0046] Terman, M. , Macchi, M. M. , Goel, N. , Rifkin, J. B. , Terman, J. S. , & Williams, J. B. W. (2003). Diagnostic reliabiity and symptom pattern of DSM‐IV atypical features in seasonal and nonseasonal depression. Chronobiology International, 20, 1157–1159.

[fsn34417-bib-0047] Terman, M. , Rifkin, J. B. , Stewart, J. W. , & Williams, J. B. W. (1998a). Diagnostic interview for atypical depression (DIAD). New York State Psychiatric Institute. https://eprovide.mapi‐trust.org/instruments/diagnostic‐interview‐for‐atypical‐depression

[fsn34417-bib-0048] Terman, M. , Rifkin, J. B. , Stewart, J. W. , & Williams, J. B. W. (1998b). The Diagnostic Interview for Atypical Depression (DIAD): Background and Instructions for Raters . 10.13140/RG.2.2.18999.57765

[fsn34417-bib-0049] Willner, P. , Scheel‐Krüger, J. , & Belzung, C. (2013). The neurobiology of depression and antidepressant action. Neuroscience & Biobehavioral Reviews, 37(10), 2331–2371. 10.1016/j.neubiorev.2012.12.007 23261405

[fsn34417-bib-0050] World Health Organization . (2023). Depressive disorder (depression) . Newsroom Fact Sheets. Retrieved 11 July 2023. https://www.who.int/news‐room/fact‐sheets/detail/depression

[fsn34417-bib-0051] World Medical Association . (2022). WMA Declaration of Helsinki – Ethical Principles for Medical Research Involving Human Subjects . Retrieved 13 July 2023. https://www.wma.net/policies‐post/wma‐declaration‐of‐helsinki‐ethical‐principles‐for‐medical‐research‐involving‐human‐subjects/

[fsn34417-bib-0052] Xie, F. , Huang, T. , Lou, D. , Fu, R. , Ni, C. , Hong, J. , & Ruan, L. (2022). Effect of vitamin D supplementation on the incidence and prognosis of depression: An updated meta‐analysis based on randomized controlled trials. Frontiers in Public Health, 10, 903547. 10.3389/fpubh.2022.903547 35979473 PMC9376678

[fsn34417-bib-0053] Yang, H. , Gao, S. , Li, J. , Yu, H. , Xu, J. , Lin, C. , Yang, H. , Teng, C. , Ma, H. , & Zhang, N. (2022). Remission of symptoms is not equal to functional recovery: Psychosocial functioning impairment in major depression. Frontiers in Psychiatry, 13, 915689. 10.3389/fpsyt.2022.915689 35958633 PMC9360322

[fsn34417-bib-0054] Young, J. E. (1998). Young schema questionnaire short form (1st ed.). Cognitive Therapy Center.

[fsn34417-bib-0055] Young, J. E. , & Brown, G. (1994). Young schema questionnaire. In J. E. Young (Ed.), Cognitive therapy for personality disorders: A schema‐focused approach (pp. 63–76). Professional Resource Exchange.

[fsn34417-bib-0056] Zanarini, M. C. , Skodol, A. E. , Bender, D. , Dolan, R. , Sanislow, C. , Schaefer, E. , Morey, L. C. , Grilo, C. M. , Shea, M. T. , McGlashan, T. H. , & Gunderson, J. G. (2000). The collaborative longitudinal personality disorders study: Reliability of Axis I and II diagnoses. Journal of Personality Disorders, 14(4), 291–299. 10.1521/pedi.2000.14.4.291 11213787

[fsn34417-bib-0057] Zimmerman, M. , Posternak, M. A. , & Chelminski, I. (2004). Derivation of a definition of remission on the Montgomery–Asberg depression rating scale corresponding to the definition of remission on the Hamilton rating scale for depression. Journal of Psychiatric Research, 38(6), 577–582. 10.1016/j.jpsychires.2004.03.007 15458853

