# Supplementary material for: A follow‐up study with a double‐blinded, randomized controlled vitamin D supplementation trial in patients with major depressive episode (DepFuD): A study protocol and baseline characteristics
Source: Food Sci Nutr. 2024 Aug 20;12(10):8454–69. doi: 10.1002/fsn3.4417 (PMC11521731; doi:10.1002/fsn3.4417)
Supplement: Supplementary file 1 — Table S1 [file FSN3-12-8454-s001.docx]

Supplementary Table S1: World Health Organization Trial Registration Data Set (Version 1.3.1) for DepFuD

https://www.who.int/clinical-trials-registry-platform/network/who-data-set

|  | Item | Description |
| --- | --- | --- |
| 1. | Primary registry and trial-identifying number | ClinicalTrials.gov Identifier: NCT02521012 |
| 2. | Date of registration in primary registry | August 13, 2015 |
| 3. | Secondary identifying numbers | DepFuDKUH5703453 |
| 4. | Source(s) of monetary or material support | Finnish state research funding (VTR). |
| 5. | Primary sponsor | There are no sponsors. |
| 6. | Secondary sponsor(s) | There are no sponsors. |
| 7. | Contact for public queries | Maarit Pakarinen, maarit.pakarinen@pshyvinvointialue.fi, Kuopio University Hospital, Department of Psychiatry, PO BOX 100, FI-70029 Kuopio, Finland |
| 8. | Contact for scientific queries | Principal Investigator: Maarit Pakarinen, maarit.pakarinen@pshyvinvointialue.fi, Kuopio University Hospital, Mental Health and Wellbeing, PO BOX 100, FI-70029 Kuopio, Finland |
| 9. | Public title | A follow-up study with randomised, controlled vitamin D trial for depression. |
| 10. | Scientific title | A follow-up study with a double-blinded, randomised controlled vitamin D supplementation trial in patients with major depressive episode (DepFuD). |
| 11. | Countries of recruitment | Finland |
| 12. | Health condition(s) or problem(s) studied | Major depressive disorder (MDD). |
| 13. | Intervention(s) | 100 micrograms “higher-dose” of vitamin D (cholecalciferol)/day supplementation in tablets versus “lower-dose” 10 micrograms/day supplementation for six months. |
| 14. | Key inclusion and exclusion criteria | Inclusion: Eligible ages: 18 Years to 65 Years. Eligible sexes: All. Patient in outpatient treatment for depression and diagnosed as mild, moderate or severe depression, or mild, moderate or severe depressive episode of recurrent depression.  Exclusion: Psychotic depression, psychotic disorder or bipolar disorder. Disease affecting vitamin D metabolism. Pregnancy or breastfeeding. Current use of a vitamin D supplement (more than 10 µg/day) (≤10 µg/day may be used regularly). Current use of a calcium supplement (more than 1200 mg/day). |
| 15. | Study type | A clinical double-blinded, parallel-group, randomized controlled superiority trial aiming to investigate the effect of vitamin D supplementation on depressive symptoms in psychiatric adult outpatients. Phase: not applicable. Sequence generation & allocation concealment: computer-generated random numbers, stratified blocked randomization with a block size of ten. Allocation sequence is concealed in a sealed envelope until intervention analyses are performed. |
| 16. | Date of first enrolment | The pilot data collection: November 2015 |
| 17. | Target sample size | Sample size plan: a total of 191 subjects per intervention group (382 in total), and considering an estimated attrition of 20 %, a total of n=478 patients must be recruited to the study.  Number of participants that the trial has enrolled: 319. |
| 18. | Recruitment status | Participants are no longer being recruited. Patient-specific follow-up lasts six years. |
| 19. | Primary outcome | Between-group (higher-dose versus lower-dose group) differential mean change from baseline to six months in depressive symptoms as measured by the Montgomery-Åsberg Depression Rating Scale (MADRS) (range: 0-60). |
| 20. | Key secondary outcome | Secondary outcomes are other indicators of mental health and functionality (BDI, SOC, 15-D, PSS10, LS-4, LOT-R, YSQ-S2-extended, CORE-OM, TAS-20, BRCS, TADS, SCL-90, DIAD, GAF), and circulating biomarkers. Intervention assessments are conducted at baseline, three, and six months, and follow-ups at 18 months and six years post-baseline. |
| 21. | Ethics Review | The research ethics committee of the Hospital District of Northern Savo, Kuopio University Hospital (ethics approval and consent number 39/2015). |
| 22. | Completion date | Estimated Study Completion Date: October 2025. |
| 23. | Summary results | No results posted. |
| 24. | Individual clinical trial participant-level data (IPD) sharing statement | Plan to share IPD: No. |

Abbreviations: BDI: Beck Depression Inventory; BRCS: Brief Resilient Coping Scale; CORE-OM: Clinical Outcomes in Routine Evaluation Outcome Measure; DIAD: Diagnostic Interview for Atypical Depression; GAF: Global Assessment of Functioning Scale; LOT-R: Life Orientation Test - Revised 6-item version; LS-4: Life Satisfaction Scale; MADRS: Montgomery-Åsberg Depression Rating Scale; PSS10: Cohen’s Perceived Stress Scale; SCL-90: Symptom Checklist; SOC: Sense of Coherence Scale; TADS: The Trauma and Distress Scale; TAS-20: Toronto Alexithymia Scale; YSQ-S2-extended: Young Schema Questionnaire S2-extended
